# Environmental and individual factors associated with quality of life of adults who underwent bariatric surgery: a cohort study

**DOI:** 10.1186/s12955-020-01331-1

**Published:** 2020-03-30

**Authors:** Marina Dayrell de Oliveira Lima, Thales Philipe Rodrigues da Silva, Mariana Carvalho de Menezes, Larissa Loures Mendes, Milene Cristine Pessoa, Lauro Pinheiro Ferreira de Araújo, Roberto Guimarães Cabezas Andrade, Alexandra Dias Moreira D’Assunção, Bruna Figueiredo Manzo, Allana dos Reis Corrêa, Fernanda Batista Oliveira Santos, Sheila Aparecida Ferreira Lachtim, Giselle Lima de Freitas, Lucas Adailton Viana de Andrade, Marco Aurélio de Sousa, Fernanda Penido Matozinhos

**Affiliations:** 1 Postgraduate Program in Nursing, School of Nursing, Universidade Federal de Minas Gerais, Belo Horizonte, Minas Gerais Brazil; 2 Postgraduate Program in Health Sciences, Child and Adolescent Health, Faculty of Medicine, Universidade Federal de Minas Gerais, Belo Horizonte, Minas Gerais Brazil; 3 Departament of Clinical and Social Nutrition, Universidade Federal de Ouro Preto, Ouro Preto, Minas Gerais Brazil; 4 Department of Nutrition, School of Nursing, Universidade Federal de Minas Gerais, Belo Horizonte, Minas Gerais Brazil; 5 Santa Rita Hospital, Contagem, Minas Gerais Brazil; 6 Department of Maternal Child Nursing and Public Health, Universidade Federal de Minas Gerais, Belo Horizonte, Minas Gerais Brazil; 7 Basic Nursing Departament, Universidade Federal de Minas Gerais, Belo Horizonte, Minas Gerais Brazil; 8 Department of Medicine, Faculdade de Minas, Belo Horizonte, Minas Gerais Brazil

**Keywords:** Obesity, Built environment, Bariatric surgery, Quality of life

## Abstract

**Introduction:**

Obesity is a multifactorial chronic condition associated with genetic, behavioral and environmental factors. Understanding the role of the built and social environment in Quality of Life (QOL) is critical to reducing the negative impacts of the environment on health.

**Objective:**

To estimate the built and social environmental and individual factors that influence the QOL of adults who underwent bariatric surgery.

**Methods:**

A prospective cohort study conducted with adults who underwent bariatric surgery. Using longitudinal linear regression analysis, we verified the association between the domains of World Health Organization Quality of Life in version bref (WHOQOL-Bref) – General QOL and domains psychological, physical health, social relations and environment – and possible influencing factors.

**Results:**

The increase in Body Mass Index (BMI) reduces on average 0.47 points in physical domain assessment score. The increase of healthy establishments within the buffer increases on average 0.52 points in the physical domain score. Being female reduces, on average, 5.35 points in the psychological domain evaluation score. Adults who practiced less than 150 min a week of leisure-time physical activity had a 3.27 point average reduction in the social relations domain assessment score. The increase in the number of Supermarkets and Hypermarkets in the buffer increases on average 2.18 points from the Social Relations domain score.

**Conclusions:**

Individual and contextual factors were associated with the QOL of adults who underwent bariatric surgery. Although the surgery yields positive results, the maintenance of same is strongly related to changes in lifestyle, the built environment and multi-professional guidance.

## Introduction

Obesity is a multifactorial chronic condition associated with genetic, behavioral and environmental factors [1]. It is also a known risk factor for the development of several diseases, such as cardiovascular diseases, diabetes and cancer [2] and its global prevalence is high [3, 4].

Globally, World Health Organization (WHO) statistics shows more than 39% of adults aged 18 years and above were overweight in 2016, with more than 13% of individuals had obesity [3]. In Brazil, the 2019 Survellience of Risk and Protective factors for Chronic Diseases by Telephone Survey (VIGITEL) showed that 55.7% of the Brazilian population was overweight and 19.8% had obesity [4].

Obesity can be classified into three types according to Body Mass Index (BMI). Class I obesity is characterized by a BMI of 30.0 to 34.9 kg / m^2^; Class II obesity ranges from 35.0 to 39.9 kg / m^2^ and BMI ≥ 40.0 kg / m^2^ is considered class III obesity [5].

Bariatric surgery is a part of the treatment of obesity. The Ministry of Health (MH) has laid down criteria for the approval of bariatric surgery: BMI of 50 kg / m^2^; BMI of 40 kg / m^2^ with or without comorbidities and unsuccessful with longitudinal clinical treatments, and individuals with BMI > 35 kg / m^2^ with comorbidities and unresponsive to longitudinal clinical treatments [6, 7]. The longitudinal clinical treatment includes guidance and support aimed at lifestyle changes, dietary reeducation, psychological attention, prescription of physical activity and, if necessary, pharmacotherapy. Therefore, surgery is only a part of the complete and complex treatment of obesity [6, 8].

Bariatric surgery confers significant weight loss, improvement in comorbidities, and most importantly, improvement in quality of Life (QOL). In fact, quality of life is one of the major reasons why individuals opt for bariatric surgery [9, 10]. QOL can be defined as an individual’s perception of his/her position in life. It covers culture, values, goals, expectations, standards, concerns and the environment in which an individual lives [11].

Considering the intrinsic relation between individuals and the environment – which has an impact on health status and QOL [12, 13] - attention should be paid to the conditions in which people reside, study and work [14].

The urban environment is dynamic and its design should minimize risks and promote QOL [15]. Health or the adoption of healthy lifestyles is directly related to the environment, accordingly it can contribute to unhealthy choices, consequently related to the QOL of individuals [16, 17].

Studies on obesity predominantly focus on investigation of individual factors [18, 19]. Despite its importance, research on environmental factors related to obesity are underexplored in Brazil, especially as regards their effect on the QOL of individuals who underwent bariatric surgery. In addition, studies conducted in specific settings and population, such as private health institutions and individuals with obesity are scarce.

Given that the relation of biological and behavioral factors with obesity is consolidated, the environmental model needs more research attention. The evaluation of the outcomes of bariatric surgery should not be entirely focused on weight loss, complications from surgery, the length of surgery, costs of the procedure, and associated morbidity and mortality rate but also on quality of life linked to environmental factors. This is because the surgery is not only aimed at weight loss but also improvement in QOL as regards the performance of activities [6]. Thus, understanding the role of the built environment (physical aspects of the environment that was built or modified by man) and social environment (socioeconomic composition and the individual and collective living conditions of the neighborhoods) on QOL is critical to the development of effective obesity prevention and management strategies and thus reducing the negative impacts of the environment on health. In this context, the objective of this study was to estimate the built and social environmental and individual factors that influence the QOL of adults who underwent bariatric surgery.

## Methods

This is a prospective cohort study conducted with adults (older than 18 years), living in the metropolitan region (municipalities of Contagem and Belo Horizonte, state capital) of Minas Gerais - Brazil, and who underwent bariatric surgery in a private hospital from 2012 to 2014.

The cohort began in 2016 and had a sample size of 133 individuals and all adults who underwent a bariatric surgery at hospital were inclued. There were no sample losses. Data collection was performed through telephone calls to individuals at the beginning of each year, with the help of a structured questionnaire based on socioeconomic, clinical, nutritional and lifestyle variables.

All stages of data collection were performed by previously trained researchers.

The same questionnaire was used in the 3 years of collection, so the variables that did not change over time were not questioned more than once.

The QOL of individuals (outcome variable) was assessed by the World Health Organization’s Quality of Life questionnaire, in its BREF (abbreviated) version (WHOQOL - bref), validated for the Brazilian population [20]. The questionnaire considers the two last weeks lived by the interviewee and consists of 26 questions or facets, of which 24 are divided in 4 domains: psychological health, physical health, social relations and environment. The instrument also presents two general QOL questions: the perception of QOL and satisfaction with health [11, 20]. In this study, the analysis of QOL was performed using General QOL (perception of QOL and satisfaction with health) and the four WHOQOL-BREF domains (psychological, physical health, social relations and environment) over the 3 years of the cohort study.

For response analysis, the values ​​of all domains are evaluated separately and transformed on a scale from 0 to 100. The score follows a positive scale. Thus, the closer to 100 the score, the better the quality of life in that domain.

For this study, independent individual variables were presented in units/categories, which include sociodemographic, economic, clinical and behavioral variables.

The environmental variables were obtained from the Brazilian government database which provides information on food sale outlets registered according to the National Classification of Economic Activities (CNAE), a standard board which assigns codes of economic activity and defines criteria used by Taxation authorities in Brazil [21], and the studied municipalities.

Geocoding of the full addresses of the environment variables was performed with the ggmap package in R, version 3.4.3. In this process, geographical coordinates (latitude and longitude) of food outlets, locations where physical activity are practiced as well as residence of individuals were located on a map.

The classification of the food environment was based on the predominant type of food available in the food outlet, predominant processing degree of marketed foods and the direction of the association of point of sale type with food consumption and/or weight gain [21, 22].

The classification was as follows: Mixed outlets - predominantly marketed ultra-processed foods concomitantly fresh and minimally processed (restaurants and bakeries), unhealthy outlets - where predominantly ultra-processed foods are sold (minimarkets, grocery stores and warehouses; retail shops that sell sweets, candies, chocolates and the like; snack bars, tea houses and juice bars and similar outlets), healthy food outlets - where predominantly fresh and minimally processed foods are traded (retail butcher shops; fishmongers; vegetable and fruit stores) and supermarkets and hypermarkets - category analyzed in isolation, given the lack of consensus in the literature about the real influence on individuals’ consumption attitudes, considering the wide range of foods available in these spaces (large outlets that sell a variety of food products in addition to having a bakery, meat, cold cuts, fruit and vegetables sections) [21, 22].

The places used for the practice of physical activities were analyzed with the availability of public and private spaces for this exercise.

Finally, the category Bars and Beverages (retailers, bars and other points of sale specialized in serving beverages) was also analyzed separately because there is no agreement in the literature on the predominance of marketed foods, taking into account the variety of products sold [23]. The social environment was assessed using the average neighborhood income. The neighborhood income was assessed based on the average monthly income per capita of the individuals' homes and was categorized into tertiles. Information on neighborhood income and population was obtained from the 2010 of Instituto Brasileiro de Geografia e Estatística demographic census database, referring to the geographical limits of the urban census sectors in Belo Horizonte and Contagem, Minas Gerais, Brazil.

To evaluate the built and social environment of the participants, the concept of neighborhood was created with buffers. This study considered neighborhood as being 500 m radius buffer, with the individuals residence being the centroid. This radius was established based on the fact that walking time may vary from 10 to 20 min [24].

The study population was described and the estimates were presented in proportions (%) with 95% CI. For the quantitative variables, the data were presented as means and standard deviation (SD) after the verification of symmetry by the Shapiro-Wilk test.

To verify the association between the WHOQOL-bref QOL domains and possible influencing factors, we used longitudinal linear regression analysis considering intra-individual correlation since the adults were being monitored over a period of time. Five independent multiple longitudinal linear regression models were constructed consisting of the four QOL domains and general QOL. The level of statistical significance at all phases of the study was 5%.

A verbal informed consent was provided by the individuals because of the data collection method, telephone interview. The study was approved by the Research Ethics Committee of Universidade Federal de Minas Gerais, under number CAAE-52657115.2.0000.5149.

The interviewees were informed about the confidentiality and anonymity of the data and that they would be used only for research purposes.

Participation of the adults was voluntary.

## Results

Table 1 shows the sociodemographic, clinical and behavioral profiles of the individuals at baseline, who underwent bariatric surgery. Note that the total number of variables may vary due to some individuals not responding to certain variables.

**Table 1:** Sociodemographic, clinical and behavioral profile of adults who underwent bariatric surgery, Cottage and Belo Horizonte, Minas Gerais – 2016 (Baseline)

	n(%)
**SOCIODEMOGRAPHIC PROFILE**	
**Sex**	
Male	22 (16,54)
Female	111 (83,46)
**Age range**	
18 to 40 years	85 (63,91)
41 to 59 years	45 (33,83)
> 60 years	3 (2,26)
**Level of education**	
University	32 (24,06)
High school	78 (58,65)
Elementary school	13 (9,77)
Primary education	10 (7,52)
**Marital status**	
Does not live with partner	39 (29,32)
Lives with partner	94 (70,68)
**Income**	
Up to 1 minimum wage	4 (3,17)
1 to 3 minimum wage	67 (53,17)
3 to 5 minimum wage	37 (29,37)
More than 5 minimum wage	18 (14,29)
**Self-declared skin color**	
White	52 (39,10)
Black	18 (13,53)
Brown	58 (43,61)
Yellow	5 (2,46)
**CLINICAL PROFILE**	
BMI*	27,60 (4,38)
**Guidance of psychologist or psychiatrist**	
No	126 (94,74)
Yes	7 (5,26)
**Guidance of nutritionist**	
No	127 (95,49)
Yes	6 (4,51)
**Systemic Arterial Hypertension**	
Yes	7 (5,26)
No	126 (94,74)
**Diabetes*mellitus***	
Yes	3 (2,26)
No	130 (97,74)
**BEHAVIORAL PROFILE**	
**Physical activity practice at leisure time**	
> 150 min per week	57 (42,86)
< 150 min per week	76 (57,14)
**Average screen time per day (TV)**	
Does not watch TV	4 (3,01)
< 1 h	25 (18,80)
1 to 3 h	68 (51,13)
3 to 5 h	31 (23,31)
> 5 h	5 (3,76)
**Alcohol consumption**	
Yes	52 (39,39)
No	80 (60,61)
**Habit of smoking**	
Yes	9 (6,82)
No	111 (84,09)
Former smoker	12 (9,09)

Source: Authors

Notes: 95% CI = 95% Confidence Interval; * Average (± SD)

The sample consisted predominantly of females, representing 83.46% (*n* = 111) of the total individuals, with 63.91% (*n* = 85) in the age group of 18 to 40 years, 70.68% (*n* = 94) lived with a partner, 58.65% (*n* = 78) with high school graduates, 53.17% (*n* = 67) with an income of 1 to 3 minimum wages and 43.61% (*n* = 58) self-declared brown skin (Table 1).

In relation to clinical profile, mean postoperative BMI was 27.60 kg/m^2^, 94.74% (*n* = 126) of the sample did not have follow-up guidance of a psychologist or psychiatrist after bariatric surgery, 95.49% (*n* = 127) did not have follow-up guidance of a nutritionist, 94.74% (*n* = 126) were not diagnosed with Systemic Arterial Hypertension (SAH) after bariatric surgery and 97.74% (*n* = 126) were not diagnosed with DM after bariatric surgery (Table 1).

As regards behavioral profile, 57.14% (*n* = 76) of the participants practiced physical activity less than 150 min per week. Regarding average screen time per day (television), 51.13% (*n* = 68) reported watching television 1 to 3 h a day. In addition, 60.61% (*n* = 80) did not consume alcohol and 84.09% (*n* = 111) were not smokers (Table 1).

A predominance of unhealthy outlets were found within 500 m buffer from individuals home. An average of 11.03 unhealt hy outlets per buffer was found and 97.74% (*n* = 126) of the adults were close to at least 1 unhealthy outlet. With the defined buffer, 65.41% (*n* = 87) of individuals had at least 1 location for physical activity practice (Table 2).

**Table 2:** Neighborhood buffer profile (500 m) of adults who underwent bariatric surgery, Contagem and Belo Horizonte, Minas Gerais - 2018

	Average (±DP)	Adults who own at least 1 establishment
**Outlet**		
Mixed	9,08 (7,82)	126 (94,74%)
Unhealthy	11,03 (9,88)	130 (97,74%)
Healthy	4,92 (4,14)	120 (90,23%)
Bar and beverages	6,69 (5,28)	125 (93,98%)
Supermarkets and hypermarkets	0,68 (1,01)	53 (39,85%)
Places used for the practice of physical activity	1,45 (1,54)	87 (65,41%)

Source: Authors

**Fig. 1:**
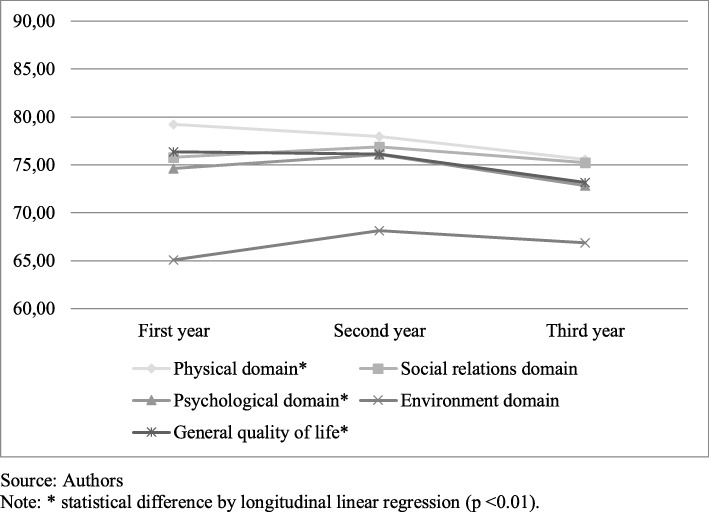
General Quality of Life and WHOQOL-BREF domains during the cohort study

From the QOL analysis, there was a significant decrease over the 3 years in the scores of physical and psychological domains and self-perception of general quality of life (Fig. 1). The bivariate analysis of the individual factors associated with the general QOL and the four WHOQOL-BREF domains, described in the Table 3, showed an association of sex (*p* = 0.013), BMI (*p* = 0.011), hypertension diagnosis after bariatric surgery (*p* = 0.025) and physical activity practice at leisure time (*p* = 0.023) with general QOL. The physical domain was associated with BMI (*p* = 0.023). The variables sex (*p* = 0.044) and physical activity during leisure time (*p* = 0.033) were associated with the social relations domain. In the psychological domain, an association was observed with BMI (*p* = 0.048) and DM diagnosis (*p* = 0.002). The environment domain showed an association with age group (*p* = 0.019) and income (*p* = 0.009) (Table 3).

**Table 3:** Bivariate analysis of individual factors according to General Quality of Life and domains of WHOQOL-BREF, Contagem and Belo Horizonte, Minas Gerais – 2016 to 2018

	General Quality of life		Quality of life domains according to WHOQOL-BREF							
			Physical		Social relations		Psychological		Environment	
	Crude Model		Crude Model		Crude Model		Crude Model		Crude Model	
	Beta (CI95%)	***p***-value*	Beta (CI95%)	***p***-value*	Beta (IC95%)	***p***-value*	Beta (CI95%)	***p***-value*	Beta (CI95%)	***p***-value*
**SOCIODEMOGRAPHIC PROFILE**										
**Sex**		**0,013**		0,234		**0,044**		0,135		0,068
Male	1		1		1		1		1	
Female	−4,89 (−8,76;-1,02)		−3,35 (−8,89; 2,17)		−6,47 (− 12,80; −0,14)		− 4,17 (− 9,65; 1,30)		− 4,05 (− 8,40; 0,30)	
**Age range**		0,432		0,360		0,402		0,114		**0,019**
18 to 40 years	1		1		1		1		1	
41 to 59 years	−1,11 (−3,75;− 1,52)		-1,82 (−5,64; 1,99)		−3,16 (−8,22; 1,89)		− 3,77 (− 7,84; 0,29)		− 3,50 (− 6,99; − 0,01)	
> 60 years	1,31 (− 3,18;5,81)		−4,62 (− 11,41; 2,17)		0,07 (−6,87; 7,01)		0,17 (− 4,58; 4,92)		4,94 (− 1,96; 11,85)	
**Level of education**		0,943		0,126		0,699		0,756		0,180
University	1		1		1		1		1	
High school	0,18 (−3,48;3,86)		4,90 (−0,06; 9,87)		2,10 (−4,14; 8,35)		0,97 (−4,26; 6,21)		−1,82 (−5,49; 1,83)	
Elementary school	−1,62 (−8,13;4,87)		−0,28 (−9,86; 9,28)		− 1,84 (− 12,90; 9,21)		−4,69 (− 16,26; 6,88)		−10,00 (− 19,07; − 0,93)	
Primary education	−0,36 (− 5,35;4,63)		−1,08 (− 10,37; 8,21)		4,31 (− 4,82; 13,44)		−1,10 (− 10,66; 8,44)		−2,24 (− 9,67; 5,17)	
**Marital conjugal**		0,895		0,615		0,252		0,722		0,701
Does not live with partner	1		1		1		1		1	
Lives with partner	−0,22 (−3,63;3,17)		− 0,99 (−4,88; 2,89)		3,00 (−2,14; 8,15)		− 0,69 (− 4,54; 3,14)		− 0,61 (− 3,75; 2,52)	
**Income**		0,569		0,472		0,880		0,735		**0,009**
Up to 1 minimum wage	1		1		1		1		1	
1 to 3 minimum wage	2,42 (−1,66;6,51)		4,91 (−1,56; 11,39)		1,79 (−6,23; 9,82)		2,92 (−3,60; 9,45)		3,67 (−2,47; 9,82)	
3 to 5 minimum wage	2,55 (−1,75;6,86)		4,74 (−2,02; 11,51)		2,37 (−6,10; 10,86)		3,18 (−3,63; 10,01)		5,12 (−1,50; 11,74)	
More than 5 minimum wage	3,33 (− 1,28;7,96)		3,63 (− 3,57; 10,84)		0,52 (−8,74; 9,78)		4,03 (− 3,02; 11,10)		9,45 (2,29; 16,62)	
**Self-declared skin color**		0,668		0,553		0,819		0,182		0,612
White	1		1		1		1		1	
Black	−2,42 (−6,82;1,96)		−3,17 (−9,98; 3,63)		−1,01 (−8,92; 6,88)		−0,32 (−7,61; 6,97)		−2,01 (−7,22; 3,20)	
Brown	0,17 (−3,02;3,37)		1,37 (−2,91; 5,65)		2,15 (−3,55; 7,86)		4,51 (−0,46; 9,49)		1,47 (−2,38; 5,34)	
Yellow	−1,78 (− 16,11;12,54)		−4,15 (− 21,64; 13,34)		0,63 (− 18,93; 20,20)		− 4,25 (− 22,32; 13,81)		1,44 (− 11,95; 14,84)	
**CLINICAL PROFILE**										
BMI*	−0,34 (− 0,60;− 0,07)	**0,011**	− 0,46 (− 0,87; − 0,06)	**0,023**	−0,09 (− 0,68; 0,49)	0,756	−0,46 (− 0,91; − 0,00)	**0,048**	−0,00 (− 0,37; 0,37)	0,986
**Guidance of psychologist or psychiatrist**		0,287		0,219		0,509		0,267		0,770
Yes	1		1		1		1		1	
No	3,81 (−3,21; 10,84)		7,58 (−4,52; 19,70)		4,90 (−9,64; 19,44)		5,87 (−4,51; 16,26)		1,15 (−6,59; 8,90)	
**Guidance of nutritionist**		0,792		0,732		0,834		0,311		0,854
No	1		1		1		1		1	
Yes	-0,89 (−7,56;5,77)		−1,73 (− 11,69; 8,22)		−1,35 (− 14,01; 11,31)		4,52 (−4,23; 13,29)		0,95 (−9,29; 11, 21)	
**Systemic Arterial Hypertension**		**0,025**		0,184		0,781		0,698		0,724
Yes	1		1		1		1		1	
No	3,79 (0,47;7,11)		4,77 (−2,28; 11,83)		1,19 (−7,25; 9,64)		−0,69 (−4,21; 2,82)		0,77 (−3,52; 5,06)	
**Diabetes*mellitus***		0,472		0,199		0,667		**0,002**		0,791
Yes	1		1		1		1		1	
No	2,62 (−4,53;9,79)		−7,32 (−18,49; 3,85)		−1,91 (− 10,62; 6,80)		−6,87 (− 11,25; −2,48)		− 0,75 (− 6,36; 4,84)	
**BEHAVIORAL PROFILE**										
**Practice of physical activity atleisure time**		**0,023**		0,080		**0,033**		0,901		0,927
> 150 min per week	1		1		1		1		1	
< 150 min per week	−2,27 (−4,22;-0,31)		−2,51 (−5,32; 0,29)		−3,35 (−6,44; −0,26)		− 0,18 (− 3,09; 2,72)		0,11 (− 2,47; 2,71)	
**Average screen time per day (TV)**		0,139		0,446		0,157		0,128		0,945
Does not watch TV	1		1		1		1		1	
< 1 h	3,45 (0,18;6,73)		3,41 (−2,55; 9,39)		6,27 (−0,92; 13,47)		3,83 (−1,55; 9,23)		−1,23 (−6,40; 3,94)	
1 to 3 h	3,36 (0,00;6,72)		1,95 (−3,86; 7,77)		6,45 (0,11; 12,80)		5,18 (− 0,11; 10,47)		0,05 (−5,04; 5,15)	
3 to 5 h	3,72 (− 0,40;7,86)		1,43 (−4,88; 7,76)		2,46 (−4,66; 9,60)		1,71 (−4,53; 7,96)		− 0,15 (− 6,00; 5,70)	
> 5 h	1,13 (− 4,34;6,60)		−2,65 (− 11,16; 5,85)		4,46 (− 4,62; 13,56)		4,37 (− 2,18; 10,93)		−0,63 (−7,73; 6,46)	
**Alcohol consumption**		0,783		0,474		0,766		0,748		0,902
Yes	1		1		1		1		1	
No	−0,44 (−3,65;2,75)		−1,59 (−5,96; 2,77)		−0,79 (−6,03; 4,44)		0,76 (−3,94; 5,47)		−0,23 (−4,06; 3,58)	
**Habit of Smoking**		0,063		0,184		0,095		0,058		0,440
Yes	1		1		1		1		1	
No	5,72 (0,95; 10,50)		4,55 (−0,32; 9,43)		9,34 (0,03; 18,64)		6,43 (0,69; 12,17)		3,24 (−1,76; 8,26)	
Former smoker	5,26 (0,47; 10,06)		3,61 (−1,37; 8,59)		7,03 (−2,08; 16,15)		6,87 (1,21; 12,54)		2,83 (−2,68; 8,35)	

Source: Authors

Note: Bold numbers = statistical significance

From the multivariate analysis, the general QOL model showed no statistical significance, indicating that the environmental and individual variables had no association with overall QOL.

The WHOQOL-BREF QOL physical domain model (Table 4) shows that the increase in BMI is associated with a reduction of, on average, 0.47 points in this assessment score, adjusted for other variables of the model.

**Table 4:** Final WHOQOL-BREF quality of life model by domain, Contagem and Belo Horizonte, Minas Gerais – 2016 to 2018

	PHYSICAL*	PSYCHOLOGICAL	SOCIAL RELATIONS*	ENVIRONMENT*
	Beta (CI95%)	Beta (CI95%)	Beta (CI95%)	Beta (CI95%)
**Sex**	−3,70(−8,77; 1,35)	−5,35**(−9,76; −0,94**)	−4,78(− 10,92; 1,36)	−2,87(−6,88; 1,12)
**Age**				
18 to 40 years (youth)	1	1	1	1
41 to 59 years (adult)	−1,02(−4,91; 2,86)	0,26(−3,68; 4,21)	−3,03(− 8,01; 1,93)	−2,80(−6,16; 0,54)
> 60 years (older adult)	− 2,08(− 9,14; 4,97)	2,84(−1,95; 7,63)	1,32(−5,84; 8,50)	4,69(− 1,76; 11,15)
**Level of education**				
University	1	1	1	1
High school	4,76(−0,57; 10,10)	−0,09(−4,22; 4,04)	2,24(−3,59; 8,08)	−0,46(− 4,17; 3,23)
Elementary school	−1,48(−11,02; 8,05)	− 4,10(− 13,08; 4,87)	0,12(− 10,07; 10,33)	−9,04**(− 17,26; − 0,81**)
Primary education	−0,49(− 10,40; 9,40)	−1,21(−7,96; 5,54)	4,89(− 4,21; 14,00)	0,36(− 6,73; 7,45)
**BMI**	−0,47**(− 0,91; − 0,04**)	−0,33**(− 0,64; − 0,01**)	–	–
**Diabetes*mellitus***				
Yes	–	1	–	–
No	–	5,53(− 3,35; 14,42)	–	–
**Habit of Smoking**				
Yes	–	1	–	–
No	–	5,77(−0,25; 11,81)	–	–
Former smoker	–	6,50 (**0,70; 12,30**)	–	–
**Income**				
Up to 1 minimum wage	–	–	–	1
1 to 3 minimum wage	–	–	–	4,67(−1,53; 10,89)
3 to 5 minimum wage	–	–	–	4,70(−1,62; 11,03)
More than 5 minimum wage	–	–	–	8,13 (**1,37; 14,88**)
**Practice of physical activity at leisure time**				
> 150 min per week	–	–	1	–
< 150 min per week	–	–	− 3,27(**−6,37; −0,18**)	–
**Healthy outlets**	0,52(**0,03; 0,99**)	–	–	0,20(−0,17; 0,58)
**Unhealthy outlets**	−0,17(− 0,47; 0,12)	–	–	–
**Bar and beverages**	−0,34(− 0,84; 0,14)	–	–	–
**Supermarkets and hypermarkets**	–	–	2,18 (**0,19; 4,16**)	–

Note:* model adjusted by the social environment variable; Source: Authors

It was also evidenced, in this model, each additional healthy outlet within 500 m buffer radius from adults home is associated with the increase average of 0.52 points on physical domain score, adjusted for other variables of the model.

The WHOQOL-BREF QOL psychological domain model (Table 4) shows that being female correlates with a reduction of, on average, 5.35 points in this assessment score, adjusted for other variables in the model.

It was also found that the increase in BMI is associated with a reduction of, on average, 0.33 points in the psychological domain assessment score, adjusted for other variables in the model.

From the WHOQOL-BREF QOL Social Relations domain model (Table 4), individuals who practiced physical activity less than 150 min per week had an average score reduction of 3.27 points compared to those who practiced more than 150 min per week.

In addition, each additional supermarkets and hypermarkets within 500 m buffer radius from adults home is associated with the increase average of 2.18 points in assessment score of quality of life as regards social relations domain, adjusted for other variables in the model.

Finally, the analysis of the WHOQOL-BREF Quality of Life Environment domain model (Table 4) showed that having elementary education is associated with a reduction of, on average, 9.04 points in this assessment score compared to adults who are high school graduates. Individuals with an income of more than 5 minimum wages increased their score by 8.13 points, compared to those with an income of up to 1 minimum wage.

## Discussion

This study revealed that environmental and individual factors are associated with almost all the domains of quality of life of individuals who underwent bariatric surgery. The predictors of a better QOL included sociodemographic (being a man, more educated), behavioral (to practice more physical activity) and clinical (lower BMI) profile, in addition to environmental aspects (more healthy outlets and more supermarkets).

As expected, the clinical profile of the adults was predominantly female. Women usually seek bariatric surgery because they are not satisfied with their physical appearance and also because of associated health problems, confirming that the pathological clinical condition is linked with obesity [25, 26]. In addition, slimness is most often associated with females due to body dissatisfaction and appearance that is incompatible with that of society, compromising relationships and activities [27].

The results of this study also show a decrease in the score of physical, psychological domains and self-perception of general QOL from 1 year to the other. Studies confirm the positive impact of the surgical procedure on individuals QOL and long term evaluation has shown that the positive outcomes of the surgery are maintained at least 1 year or up to 2 years post-surgery, and may tend to disappear after this period [28, 29].

The physical domain is associated with the basic needs of human being, physical pain, energy for daily activities, locomotion, sleep and rest, the ability to perform daily activities and work. The psychological domain is related to the frequency of negative feelings, ability to concentrate, acceptance of body image, appearance and self-esteem. Such factors are particularly important for QOL and may provide information on what motivates obese adults to opt for bariatric surgery [30, 31].

In this study, increase in BMI reduced the score of both domain, physical and psychological. A high BMI is related to low or lack of self-acceptance, increased stress level, decreased self-esteem and humor and depression, all of which are reflected in QOL deficits [7].

The results of this study also highlight the built environment as an opportunity or barrier for proper and healthy eating which consequently affects QOL. Healthy eating is only possible in food environments that promote access to adequate food and necessary living conditions [17]. The adoption of healthy lifestyles, including the consumption of adequate foods, is a fundamental requirement for QOL, as it is associated with health promotion and reduction in the incidence of NCD [32].

The food environment influences access to healthy and unhealthy foods and is related to consumption. Access to healthy food is positively influenced by the type of food intake. Increased number of outdoor food markets and supermarkets may promote better access to healthy food [33].

Being female is an important predictor of worse psychological QOL domain score, explained by the fact that women are more concerned about health [34]. Studies also indicate that women are more prone to depressive symptoms, which may negatively influence QOL [35]. Another explanation for the worse QOL observed in the female adults is the high number of hours dedicated to the home and work outside the home, social, economic, political burdens and cultural inequalities [36].

There is also an obsessive search for a standard of beauty, which often ends up blurring the thin line between care that benefits the body and the onset of disease. Nowadays, current cultural patterns bring a current that even individuals with healthy biotypes perceive their weight beyond the healthy, directly affecting the perception of body image [37]. In Brazil, the predominant aesthetic culture, the body, especially the young, “the standard”, “sexy” and especially the “thin” is considered a means of social ascension, as well as an important capital in the labor and marriage market [38].

Another possible explanation for this greater female demand is justified by cultural factors, as women are more predisposed to seek clinical care, favoring the diagnosis of diseases [26, 27].

Studies support the idea that individuals who underwent bariatric surgery usually do not create a direct relationship with BMI, but with perceived image of obesity, which is not necessarily related to the individual’s actual weight. Therefore, although there is rapid weight loss due to the surgery, some individuals show greater difficulty in observing another body pattern [31, 39].

In this study, the WHOQOL-BREF social relationship domain score of individuals who practiced less than 150 min of physical activity per week was lower compared to those who practiced more than 150 min. Physical activity can directly affect social development. The practice of physical activity is associated with lower social isolation and greater social interaction [40]. It also contributes to good physical condition, a precious tool for the improvement of QOL [41].

A positive association between increase in the number of supermarkets and hypermarkets within 500 m radius buffer of individuals homes and the evaluation of the QOL Social Relations domain was observed.

Neighborhoods with a greater number of large supermarkets can provide greater social interaction between neighbors and friends. The built environment determines access to public spaces and adequate paving, favoring greater opportunities for leisure, practice of physical activities and social interaction related to healthier lifestyles [42].

There is also a relationship between education and income with the environment domain score of QOL.

Income and level of education are considered subjective indicators of QOL, factors that assist in the provision of personal and collective needs [43]. Individuals with low levels of education, lower family income, and social vulnerability are more likely to be exposed to factors that risk their QOL. Moreover, geographical segregation which concerns the separation of social groups within a given space, highlights the fact that individuals with better socioeconomic status reside in urban spaces that present better infrastructure and safety conditions [43], factors that compose the environment domain of QOL. Education, an important factor for increase in income, is related to infrastructure and opportunities in a locality. Better-structured neighborhoods tend to encourage healthier behavior, as they offer spaces for leisure and physical activity [44].

The study has some limitations, such as the non-assessment of QOL before the surgical procedure, as well as the use of self-reported data to assess QOL after bariatric surgery. Although we did not measure QOL before surgery, the individuals were monitored for a long postoperative period, which tends to minimize the impacts related to the lack of this data. In addition, data collection by the self-report method has been widely used as an acceptable and valid method in epidemiological studies with Brazilian adults [44].

It is also emphasized that the use of a buffer to define the neighborhood to be investigated. However, this type of information has been widely used in similar context studies and we assume no changes in buffer design occurred during the study period.

In addition, the results presented need to be interpreted with caution, since the relationship between individual, built and social environment and quality of life is complex and has other variables that can interfere in this context.

The strength of the study is the use of a large sample of adults undergoing a follow-up study after bariatric surgery in a specific health institution, a private hospital, the use of a questionnaire consisting predominantly of validated questions for the Brazilian population to investigate the study. Outcome and the investigation of the impact of environmental factors on the quality of life of individuals in Brazil, research that is scarce in developing countries.

## Conclusion

The present study demonstrated that individual and environmental factors have an impact on the QOL of adults who underwent bariatric surgery. Thus, being female, high BMI, practicing physical activity less than 150 min a week, low level of education and low income, allied to environmental factors, such as decreased number of healthy outlets and supermarkets within 500 m buffer radius from individuals homes have a negative impact on the QOL of bariatric surgery adults.

Therefore, the association of individual and contextual factors determine QOL, emphasizing the relevance of lifestyle changes and the effect of the built environment on access to places that may or may not encourage healthy eating and the practice of physical activity.

The results of this study provide important epidemiological information concerning the improvement of QOL of bariatric surgery adults. Living a healthier life related to success of the surgical procedure involves the interconnection of environmental, physical, mental and social aspects which vary from individual to individual.

The study reflects improvements in QOL and positive health impacts of bariatric surgery, although the procedure does not solve all health-related problems and difficulties. It is considered whether it is possible to equate the improvements achieved in QOL of these adults to the assessment of QOL of individuals with similar BMI but who have never needed the surgical procedure.

Moreover, it is noteworthy that individuals who have undergone bariatric surgery have specific needs as well as particular clinical and behavioral characteristics which affect the way they relate to the environment compared to individuals who have never undergone the surgery.

## Data Availability

The data is confidential and belongs to the researchers.

## Abbreviations

BMI: Body Mass Index
DM: Diabetes mellitus
MH: Ministry of Health
CNAE: National Classification of Economic Activities
NUPESV: Núcleo de Estudos e Pesquisa em Vacinação
QOL: Quality of Life
DS: Standard Deviation
VIGITEL: Survellience of Risk and Protective factors for Chronic Diseases by Telephone Survey
SAH: Systemic Arterial Hypertension
WHOQOL: World Health Organization Quality of Life
WHO: World Health Organization
